# Established and emerging new approach methodologies in neuroscience

**DOI:** 10.3389/fnins.2025.1696937

**Published:** 2025-12-15

**Authors:** Dorien Imberechts, Annelii Ny, Daniëlle Copmans

**Affiliations:** 1 Laboratory for Molecular Biodiscovery, Department of Pharmaceutical and Pharmacological Sciences, KU Leuven, Leuven, Belgium; 2 Leuven Childhood Epilepsy Center, Leuven Brain Institute, KU Leuven and UZ Leuven, Leuven, Belgium

**Keywords:** neuroscience, brain organoids, zebrafish, safety screening, drug discovery

## Abstract

The increasing need for ethical, human-relevant, and efficient alternatives to animal testing is driving the development of New Approach Methodologies (NAMs) in safety assessment and drug development. However, the inherent complexity of neurological diseases presents a significant challenge to fully replace animal models in this field. In neuroscience, a range of NAMs, from traditional 2D cell cultures to advanced brain organoids and alternative vertebrate models like zebrafish, demonstrate complementary strengths and limitations. Together, these models support translational research, including the investigation of neurodevelopment, disease, and neurotoxicity. While human and mouse brain organoids that mimic the structural and functional properties of mammalian brain tissue hold great promise, their applicability for high-throughput screening is hindered by their cost- and time-intensive nature. Complementary approaches such as embryonic and larval zebrafish models and the emerging zebrafish brain organoids provide faster, cost-effective, and scalable yet biologically relevant platforms for early-phase screening, thanks to the zebrafish’s rapid development, conserved vertebrate neuroanatomy, and proven value in toxicology. This review maps the current landscape of NAMs in neuroscience, examining approaches ranging from 2D and 3D *in vitro* systems to zebrafish models. It highlights the advantages and challenges of the different models, including a comparison of human, mouse, and zebrafish brain organoids, and outlines the future directions for integrating these complementary systems into robust, efficient, and ethically responsible pipelines for both early-phase toxicity testing and drug discovery.

## Introduction

The fields of toxicology and drug development are undergoing a pivotal transformation, driven by the need for more ethical, human-relevant and predictive testing systems in accordance with the 3Rs principle (i.e., replacing, reducing, and/or refining animal studies) (Krewski et al., 2010; Kavlock et al., 2009; Punt et al., 2020; Schmeisser et al., 2023). Traditional animal models, such as rodents, have been indispensable for decades, offering critical insights into disease mechanisms and safety assessment. Undoubtedly, they continue to play a vital role in advancing our understanding today. However, given the ethical concerns, as well as the significant time and cost associated with animal testing, there is a growing incentive to reduce animal use where possible, without compromising scientific rigor and safety. This has accelerated the development of New Approach Methodologies (NAMs) (Turner et al., 2023), being alternative approaches that minimize or eliminate animal use and may complement or even improve the predictive power for human health outcomes, although, in some cases, further validation is needed.

The development and implementation of effective and reliable NAMs is also becoming necessary to meet legal regulatory requirements mandating the use of scientifically valid non-animal testing methods (European Parliament and Council, 2009; European Parliament and Council, 2006; OECD, 2022). In recent years, legislation and regulatory guidance from agencies such as the US Food and Drug Administration (FDA) and the European Medicines Agency (EMA), along with EU policies like the ban on animal testing for cosmetics (European Parliament and Council, 2009) and the Registration, Evaluation, Authorisation and Restriction of Chemicals (REACH) regulation (European Parliament and Council, 2006), have created a strong incentive to advance alternative methods.

NAMs encompass a wide variety of innovative technologies and methodologies, each with its own strengths and limitations. These include, but are not limited to, *in silico* models, basic *in vitro* toxicity assays, monolayer 2D cell cultures, organotypic co-cultures, 3D culture systems such as patient-derived organoids, more advanced microphysiological systems (e.g., organ-on-a-chip and tissue-on-a-chip), and the use of alternative non-mammalian species, including both vertebrates [e.g., *Danio rerio* (zebrafish) and *Oryzias latipes* and *Oryzias melastigma* (medaka)] and non-vertebrates [e.g., *Daphnia magna* (water flea) and *Drosophila melanogaster* (fruit fly)] (Turner et al., 2023; Schneider et al., 2021).

Among these, human organoids have emerged as one of the most promising *in vitro* tools in modern biomedical research (Kim et al., 2020; Matsui and Shinozawa, 2021). Brain organoids in particular, represent a breakthrough for neuroscience and neurotoxicology (Lancaster et al., 2013), enabling disease modeling, the study of human neurodevelopment, and responses to chemical exposure (e.g., neurotoxicants and candidate therapeutic compounds in development) in a more physiologically relevant context than traditional 2D cultures (Cao, 2022; Fan et al., 2022; Lei et al., 2024). However, despite these advantages, human brain organoids are not without limitations. Their production is time-consuming and resource-intensive, often requiring months to reach maturation stages suitable for experimentation (Qian et al., 2019). Compared to standard cell lines, brain organoids require specialized media, growth factors, and labor-intensive protocols, which raise costs considerably, often approaching those of small-scale animal models such as mice and significantly exceeding those of aquatic model organisms like zebrafish. Additionally, reproducibility between organoid batches can vary (Maisumu et al., 2025), and achieving complete representation of human brain complexity, such as vasculature (Lancaster et al., 2013; Cakir et al., 2019), microglia (Ormel et al., 2018; Sabate-Soler et al., 2022), and regional specification, remains challenging. These constraints limit their utility for large-scale, high-throughput screening purposes, posing a bottleneck for early-phase toxicity testing and drug discovery efforts where speed and scalability are critical.

Mouse organoids have emerged as a complementary model to human organoids (Marshall and Mason, 2019). Stem cell-derived mouse organoids are valuable for optimizing protocols to generate brain region-specific structures. Extensive *in vivo* data on mouse brain development, together with detailed analyses of genetic mutants, provide critical benchmarks for validating organoids as tools to study the genetics of neural development (Lloyd-Davies Sánchez et al., 2024). Moreover, they offer a practical means to reduce, or potentially replace, the use of animals in research on normal brain development. However, mouse brain organoids are costly and relatively slow (several weeks) to generate. In addition, they lack full tissue maturity and are not well-suited to high-throughput studies (Medina-Cano et al., 2025). As a result, their utility for disease modeling still largely remains experimental (Marshall and Mason, 2019).

Zebrafish are already well established in toxicology research (Bambino and Chu, 2017; Guo, 2009; Hoffmann et al., 2021; MacRae and Peterson, 2015; Nishimura et al., 2016; Rosa et al., 2022; Sipes et al., 2011; Zhao et al., 2024) due to their rapid development, optical transparency during embryogenesis, and genetic tractability (He et al., 2006; Howe et al., 2013; Kily et al., 2008; Kimmel et al., 1995; Mueller and Wullimann, 2003). They not only facilitate the early identification of neurotoxicity before advancing compounds to more costly stages of drug development, but also enhance the ethically favorable, biologically relevant, cost-effective, and scalable assessment of potential environmental neurotoxicants, such as industrial chemicals. This dual utility aligns closely with the One Health framework (FAO, UNEP, WHO, and WOAH, 2022), which emphasizes the interconnectedness of human, animal, and environmental health, supporting a more holistic strategy for safeguarding health across species and ecosystems. Importantly, essential features of vertebrate brain organization are preserved, and zebrafish share many conserved pathways of neurodevelopment, neurotransmission, and toxicity responses with humans (Guo, 2009). This species is now considered a powerful complementary model in neuroscience (Guo, 2009), offering valuable insights into brain development, neural circuitry, and neurological diseases such as epilepsy (De Meulemeester et al., 2024; Kalueff et al., 2014), and serving as a powerful tool in drug discovery and development initiatives (Sourbron et al., 2017; Thevissen et al., 2025; Baraban et al., 2013). Building on this foundation, recent advances in stem cell and developmental biology enabled the generation of zebrafish brain organoids (Zilova et al., 2021), offering a novel *in vitro* platform that combines organoid technology with the practical advantages of the zebrafish model. Zebrafish brain organoids develop significantly faster than their human and mouse counterparts and can be produced at a large scale using relatively simple protocols. While they cannot fully replicate human brain complexity (Mueller and Wullimann, 2003; Mueller and Wullimann, 2009), their potential as a faster and more scalable system for high-throughput screening could become strategically important and advantageous as a relevant intermediate model, especially when used in combination with human-derived systems for validation.

This review maps the evolving landscape of NAMs in neuroscience with a focus on organoid technology and zebrafish models. Their strengths and limitations for neurodevelopmental studies, disease modeling, and neurotoxicity screening are critically assessed and compared to *in vivo* vertebrate models. Moreover, future directions are proposed for integrating these complementary systems into robust, efficient, and ethically responsible pipelines for both early-phase toxicity testing and drug discovery and development.

### The regulatory paradigm shift

In recent years, regulatory agencies worldwide such as the EMA, the European Chemicals Agency (ECHA), US-FDA and US Environmental Protection Agency (EPA) have gradually explored and promoted the paradigm shift toward using alternative methods for preclinical research to provide reliable and predictive data without animal studies (European Parliament and Council, 2009; European Parliament and Council, 2006; OECD, 2018; Avila et al., 2020). The development and implementation of NAMs gained momentum after the EU banned animal testing for cosmetics in 2013 (European Parliament and Council, 2009; Parish et al., 2020), especially for toxicological endpoints such as skin and eye irritation. Momentum further increased with the introduction of REACH, which created a strong demand for alternative methods to assess the safety of large numbers of chemicals (European Parliament and Council, 2006). These legislative milestones not only accelerated the development of NAMs but also challenged regulators to establish clear pathways for their acceptance and integration into risk assessment frameworks. As part of this effort, the Organisation for Economic Co-operation and Development (OECD) played a key role by developing and updating internationally recognized Test Guidelines that incorporate NAMs, helping to harmonize validation standards and facilitate regulatory acceptance across countries. Consequently, academia, industry and governmental institutions made international collaborative efforts to develop a wide variety of NAMs and to validate their reproducibility, accuracy and predictivity (Turner et al., 2023; Avila et al., 2020). Such initiatives include the EU Horizon Europe and Innovative Health (European Commission, 2021) and EU’s Next Generation Risk Assessment (NGRA) program (Pallocca et al., 2022). In addition, each year, the EU Reference Laboratory for alternatives to animal testing (EURL ECVAM) publishes an overview of the progress of research projects in the EU relating to NAM development (EURL ECVAM Status Report 2024) (Baccaro et al., 2025). Together, these changes represent a paradigm shift towards modern, animal-free testing strategies that address ethical concerns while maintaining high levels of human and environmental protection.

While the pharmaceutical industry traditionally relies on animal studies due to the complex systemic safety requirements, recent scientific and regulatory initiatives, such as the FDA’s Modernization Act 2.0 (2022) (US Congress, 2021-2022), Tox21 consortium (Kavlock et al., 2009), ToxCast program (Dix et al., 2007), and the Innovative Health Initiative (IHI) in Europe (Initiative IH, n.d.), are increasingly promoting the integration of NAMs into pharmaceutical research and development. Animal studies still remain a requirement before first-in-human trials because of perceived physiological similarities between species (e.g., rodents, dogs, non-human primates), and therefore, NAMs are not (yet) envisioned as a full replacement. Instead, they are increasingly used to support early-stage efficacy and safety assessment, helping to inform and refine subsequent animal studies (Turner et al., 2023). By generating robust *in silico* and *in vitro* data early in the pipeline, NAMs will help to identify and eliminate potentially ineffective or toxic compounds before they reach animal testing. Moreover, these approaches generate valuable mechanistic and dose–response data that help prioritize candidates, optimize study design and dose selection, and ensure that subsequent animal studies are more targeted, scientifically justified, and aligned with the 3Rs principle. Overall, continued investment in NAMs will further enhance their ability to predict human toxicity, ultimately strengthening the safety and translational relevance of preclinical testing.

Reflecting this shift, regulatory guidelines such as the EMA’s Guideline on “Strategies to identify and mitigate risks for first-in-human and early clinical trials with investigational medicinal products” (van Gerven and Bonelli, 2018; European Medicines Agency (EMA), 2017) and the ICH S6 guideline on “Nonclinical safety evaluation of biotechnology-derived pharmaceuticals” (European Medicines Agency (EMA), 2011), aim to reduce clinical failures and unnecessary animal studies. They assist in species selection for safety assessments during drug development and explicitly discourage toxicity studies in non-relevant animal species. Moreover, these guidelines recommend conducting additional nonclinical testing to obtain relevant data for risk assessment, and they encourage the use of *in vitro* methods based on human-derived material. In line with this, a report by the US National Academy of Sciences (National Academies of Sciences, Engineering, and Medicine, 2023) also highlights the limitations of animal models and emphasizes that NAMs can enhance the validity of safety assessments by complementing traditional approaches.

### The current landscape of NAMs in neuroscience

The complexity of central nervous system (CNS) disorders is a key contributor to the higher failure rates of drugs targeting neurological disorders when compared to other areas of drug discovery (Gribkoff and Kaczmarek, 2017). Hence, the need for models that can capture the disease processes and the added challenge within the neuroscience field to fully replace animal testing (i.e., whole organisms) by more simple non-animal models. However, challenges in translatability arise from the unique complexity of the human brain, with species-specific features in its structure, function, and development (Sousa et al., 2017; Onishi and Shimogori, 2025). One major hurdle is the inaccessibility of human brain tissue for research. The long, complex process of human brain development and the higher-order brain processes (e.g., cognitive functions) (Kelley and Pasca, 2022) involved in complex brain disorders such as Alzheimer’s disease, Parkinson’s disease and epilepsy, are challenging to fully replicate in traditional animal models. In addition, significant differences between human and animal CNS biology such as neurotransmitter systems (Fitzgerald, 2009; Beed et al., 2020), blood–brain barrier (BBB) permeability (Hoshi et al., 2013; Uchida et al., 2013; Syvanen et al., 2009) and gene expression patterns in neurons and glial cells, can result in poor translation of efficacy and safety data from animals to humans. One example is the relatively higher proportion of cortical GABAergic interneurons compared to glutamatergic neurons in humans, in contrast to the ratios observed in non-human primates and rodents (Bakken et al., 2021; Krienen et al., 2020). Consequently, the predictive value of animal models remains imperfect, contributing to high clinical attrition rates (Sun et al., 2022).

**Figure 1:**
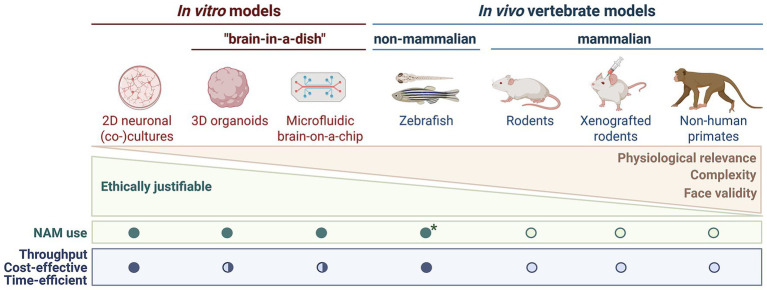
Overview of state-of-the-art preclinical models used in neuroscience. Representation of how each model, from simple 2D *in vitro* systems to complex *in vivo* xenograft models and non-human primates, differs in level of physiological relevance to human brain function, biological complexity, face validity and the extent of ethical justifiable use. High face validity includes the ability to display behavior (locomotion, sleep, tremor, seizures, etc.) and other clinically relevant observations such as cognition (memory impairment, executive dysfunction, etc.). The suitability as a new approach methodology (NAM), experimental throughput, cost-effectiveness, and time efficiency are indicated using circles: filled circle, high; semi-filled, intermediate; open circle, low. *Zebrafish embryos and larvae <120 h post-fertilization (hpf) are legally not considered laboratory animals. Figure created with Biorender.com.

Recent technological evolution in NAMs addresses some of these limitations through the integration of advanced *in silico, in vitro* and *in vivo* systems. A diverse range of NAMs are being explored to complement traditional animal studies to study specific human-relevant mechanisms, improve the prediction of neurotoxicity for human and environmental health (Cao, 2022; Fitzgerald et al., 2021), and support early-stage drug discovery and development (Robertson et al., 2025; Mehta et al., 2025). The following sections provide an overview (Figure 1) of these advancements and their potential to support more reliable, human-relevant, and ethically responsible neuroscience. Nevertheless, NAMs come with their own limitations, including the lack of complexity compared to whole organisms. Hence, they are not (yet) envisioned as a full substitute for established *in vivo* models. Instead, they serve as complementary tools that, when used together with animal studies, and in partial replacement thereof, strengthen the overall predictive power and human relevance of neuroscience research.

### Human *in vitro* approaches for brain research

In recent years, significant advancements have been made in developing *in vitro* cell models for brain research (Peng et al., 2021; Dabhekar et al., 2024). These models have become invaluable tools for investigating brain biology and pathology, substantially advancing our understanding of the brain’s complexity.

#### 2D cultures of immortalized and primary cells

Immortalized neuronal cell lines, such as the widely used human neuroblastoma SH-SY5Y cells (Encinas et al., 2000), are commonly used due to their human origin, ease of culture, ability and reproducibility to differentiate into neuron-like cells, genetic manipulability, and suitability for high-throughput screening. While they exhibit neuronal properties and express key neuronal markers (Sahin et al., 2021), they are tumor-derived and do not fully replicate the complexity or maturity of primary neurons. The latter are derived directly from brain tissue, have a limited lifespan but offer greater physiological relevance at the cellular level (Murphy-Royal et al., 2020). Cell-based transfection of DNA or RNA of interest is often performed to manipulate gene expression and to cost-effectively assess the biological and functional consequences. To more accurately mimic the intricate cellular environment of the brain, co-culture systems, combining neurons with glial cells (e.g., astrocytes, microglia, and oligodendrocytes) and/or endothelial cells (Sivandzade and Cucullo, 2018; Cucullo et al., 2011), can be used. These co-cultures enable researchers to study essential processes such as glial support for neuronal survival, synaptic regulation, and neuroinflammation. Incorporating endothelial cells provides a platform to model the complex BBB, facilitating research on neurovascular interactions and the delivery of therapeutics to the brain (Helms et al., 2016). Even though these 2D models are rather simplistic and do not fully capture the complexity of the human brain, they are invaluable tools for fundamental research (e.g., providing initial insights into cellular mechanisms) and high-throughput drug screening, including small molecules, RNAi therapeutics, and gene therapy candidates.

#### 2D iPSC-derived neural cultures

In the past two decades, advances in stem cell technology have enabled the development of more sophisticated *in vitro* models that better mimic human (patho)physiology. Human induced pluripotent stem cells (iPSCs), reprogrammed from adult somatic cells like skin-derived fibroblasts or blood mononuclear cells, possess the capacity to differentiate into all cell types found within the three germ layers (ectoderm, endoderm, and mesoderm), including various neuronal and glial cell types, similar to embryonic stem cells (Kim et al., 2011; Takahashi et al., 2007; Yu et al., 2007; Lowry et al., 2008). A wide range of differentiation protocols are established, typically involving the exposure of iPSCs to neural patterning cues and developmental signaling molecules, resulting in the reliable generation of diverse cell types such as motor neurons (Boulting et al., 2011; Dimos et al., 2008), dopaminergic neurons (Kriks et al., 2011), GABA- and glutamatergic neurons (Doorn et al., 2023; Frega et al., 2017; Mossink et al., 2022; van Hugte et al., 2023), as well as astrocytes (Krencik et al., 2011; Roybon et al., 2013). iPSC technology thereby provides an unlimited source of patient-specific cells for studying neurological disorders and disease modeling, offering particular value for diseases like epilepsy and Parkinson’s disease, characterized by significant genetic heterogeneity (Simkin et al., 2022; Klein and Westenberger, 2012). Moreover, the development of CRISPR/Cas9 technology made it possible to create isogenic iPSC lines that are identical to the original parental lines except for the specific edited sites (Seeger et al., 2017). This allows for clear genotype–phenotype correlations without confounding effects from the surrounding genetic background. Nevertheless, 2D iPSC-based disease models also have important limitations. They do not fully recapitulate the complexity of the human brain, including interactions with other organ systems, the immune system, and the surrounding microenvironment (Gattazzo et al., 2014). Additionally, variability between iPSC lines and differences in differentiation protocols across laboratories impact reproducibility and consistency, highlighting the need for standardized protocols and rigorous quality control (Volpato and Webber, 2020). Another key limitation is that iPSC-derived neurons often remain in an immature state, reducing their relevance for studying adult-onset or late-stage neurological diseases (Centeno et al., 2018). The slow maturation of neuronal cultures, on the other hand, mirrors the prolonged development of the human brain, enabling researchers to study the origins and progression of developmental diseases over time. Moreover, while iPSCs bypass many of the embryo-related ethical issues associated with ESCs, ethical challenges remain, particularly regarding donor consent, privacy, and genetic manipulation, as iPSCs retain the donor’s full genetic information (Velasco et al., 2019). Despite lacking the cellular heterogeneity, tissue architecture, and microenvironmental cues of the complex human brain, patient-specific iPSC-derived disease models serve as powerful platforms for pathomechanistic research, drug screening and personalized medicine, helping to develop tailored therapeutic strategies based on individual patient profiles (Miccoli et al., 2018).

#### 3D brain organoids

3D organoid technology bridges this gap and creates more physiologically relevant and predictive systems. Brain organoids are miniature 3D structures that arise from the self-organization, self-renewal, and differentiation of pluripotent stem cells (Lancaster et al., 2013; Bartfeld and Clevers, 2017; Fatehullah et al., 2016). They mimic key features of the developing human brain, including region-specific organization, interactions between multiple neural cell types, and the emergence of all necessary cell types for neuronal activity (Kim et al., 2020). These cells follow a human-like developmental timeline with gene expression patterns resembling those of the immature cortex (Camp et al., 2015; Pollen et al., 2014). Electrical activity of a single neuron can be measured using whole-cell patch clamp technology (Landry et al., 2023), while multielectrode arrays or calcium imaging detect activity across multiple neurons in the network simultaneously (Oliva et al., 2024). As such, brain organoids provide a promising platform for studying human brain development (e.g., neurogenesis, neuronal migration, synaptogenesis, and the creation of various brain areas), neurological disease modeling, and enhancing the predictive power of neurotoxicity testing (Bremond Martin et al., 2021; Gerakis and Hetz, 2019; Bagley et al., 2017; Trujillo and Muotri, 2018; Nzou et al., 2018). Among others, reviews by Cao (2022), Fan et al. (2022), and Lei et al. (2024), provide a comprehensive overview of the use of 3D human brain organoids in neurotoxicity evaluation. These studies show that organoid models effectively mimic human brain development and offer a valuable platform for assessing the effects of various neurotoxicants, including environmental toxins, drugs, and chemicals, on neural cells and circuits.

Despite their immense potential, human brain organoids face significant limitations that hinder their utility and ability to fully replicate the complexity of the human brain. Culturing brain organoids is time-consuming as it mirrors the natural pace of human brain development, requiring multiple differentiation stages, maturation of various cell types, and long-term growth to achieve meaningful structural and functional complexity (Qian et al., 2019). This lengthy process limits the efficiency and throughput of brain organoid production. Moreover, brain organoid protocols are often complex and lack standardization, which contributes to significant batch-to-batch variability (Maisumu et al., 2025). The sophisticated procedures also require skilled and experienced researchers to achieve consistent results, further limiting reproducibility across different labs. Additionally, generating and maintaining organoids requires expensive growth factors, specialized culture media, and advanced laboratory equipment, posing a financial barrier. These challenges collectively hinder scalability and the reliable application of brain organoids in neuroscience. Another important limitation is that human brain organoids, like other *in vitro* models, have a low face validity as they cannot model behavioral phenotypes and symptoms. For example, although organoids display seizure-like electrical activity and upregulation of molecular markers relevant to epilepsy (Samarasinghe et al., 2021) or dopaminergic neuron loss seen in Parkinson’s disease (Cui et al., 2024), they cannot replicate the motor symptoms, such as seizures or tremors, observed in patients. The same limitations apply to comorbidities such as sleep disturbances, psychological problems (e.g., depression, anxiety, and aggression), autism spectrum disorder (ASD), impaired cognition, and others.

Nevertheless, the incorporation of these more sophisticated and physiologically relevant *in vitro* models is transforming biomedical research and safety assessment in neuroscience. By improving the prediction of human responses, these models help advance health outcomes (Langhans, 2018) while simultaneously reducing the need for animal testing, in alignment with the 3Rs principle (Csukovich et al., 2022; Csukovich et al., 2022). Although the resource- and time-intensive nature of 3D brain organoids makes them low-throughput and less suitable for primary drug screening, they already complement animal studies in preclinical neuroscience research and drug development in the pharmaceutical sector, as evidenced by numerous recent reports (Matsui and Shinozawa, 2021; Beilmann et al., 2025; Beilmann et al., 2019; Pognan et al., 2023; Mina et al., 2019; Wang et al., 2022; Gjorevski et al., 2020). Their ability to detect neurotoxic or adverse effects at early stages facilitates earlier decision-making in drug pipelines, helping to avoid late-stage failures, reduce development costs, and minimize the use of both human and animal resources. This shift is further supported by international frameworks like the OECD’s Guidance Document on “Good *In Vitro* Method Practices (GIVIMP)” (OECD, 2018) and expert recommendations like those of Bal-Price et al. on “*in vitro* test readiness for developmental neurotoxicity” (Bal-Price et al., 2018), highlighting the global commitment to improve *in vitro* approaches for brain research and safety assessment. Notably, single *in vitro* tests are insufficient to fully capture the complexity of biological systems or to entirely replace animal experiments. More reliable and comprehensive data are generated by combining them with other NAMs. These combinatorial approaches, integrating *in silico, in chemico, in vitro*, and non-animal *in vivo* methods, are supported by the OECD’s framework of Integrated Approaches to Testing and Assessment (IATA) offering a more holistic and flexible strategy for safety assessment, leading to more robust and human-relevant risk evaluations (OECD, 2022; Sakuratani et al., 2018).

#### Microfluidic brain-on-a-chip platforms

A well-documented consequence of the prolonged brain organoid culture process is cell death in the organoid core, due to insufficient oxygen and nutrient diffusion (Lancaster et al., 2013; Cakir et al., 2019). To address this, dynamic culturing methods such as orbital shakers and spinning bioreactors have been introduced. The integration of organoids into microfluidic systems further enhances their physiological relevance by creating dynamic, controllable environments (Kim et al., 2020; Chauhdari et al., 2025; Cho et al., 2021). These advanced brain-on-a-chip platforms improve oxygen and nutrient delivery, facilitate waste removal, and simulate extracellular fluid flow by linking neuronal compartments through microfluidic channels. This setup more accurately mimics *in vivo* conditions and allows for the incorporation of vascular structures. As a result, *in vitro* models of the BBB are developed, enabling more sophisticated studies of neurovascular interactions, drug transport, and BBB function (Lei et al., 2024; Bergmann et al., 2018; Kawakita et al., 2022; Bell et al., 2025; Shi et al., 2020). However, the need for advanced microfluidic approaches adds to the already labor-intensive nature of the use of brain organoids, in contrast to traditional animal models, which naturally provide the full systemic complexity of a whole organism. Additionally, the spontaneous emergence of glial cells, such as astrocytes and oligodendrocytes, occurs only at later stages of organoid development (Sloan et al., 2017; Verkerke et al., 2024; Pasca et al., 2015). This delay, which reflects the natural progression from neurogenesis to gliogenesis during human embryogenesis, further prolongs the culturing timeline. Consequently, the ability to study more mature brain features and early neuron–glia interactions is limited, especially for drug discovery purposes that rely on high-throughput capacities. Nevertheless, significant research continues to focus on improving both brain organoid and brain-on-a-chip technologies, aiming to balance physiological relevance with practical scalability to make them viable for applications such as drug screening, disease modeling, and personalized medicine.

### Human neuron xenograft models

Transplantation of human neurons and brain organoids into animal brains, typically immunodeficient rodents, is a recent exploratory technique aimed at addressing the limitations of *in vitro* 2D neuronal and 3D organoid models by providing a more physiologically relevant *in vivo* context (Mansour et al., 2018; Revah et al., 2022; Xiang et al., 2017; Wilson et al., 2022; Mancuso et al., 2024; Linaro et al., 2019). Implanted neurons can integrate with the host’s vascular system, form synaptic connections with host neurons, and respond to sensory stimuli, enabling more dynamic modeling of human brain development and function. Furthermore, transplantation models overcome the fact that the gene expression profiles in 2D and 3D iPSC-derived brain cultures are altered by artificial *in vitro* conditions and remain relatively immature, thus not fully reflecting *in vivo* brain tissue. In a recent study by Revah et al. (2022), 3D human cortical brain organoids derived from hiPSCs were transplanted into the primary somatosensory cortex of immunodeficient newborn rats, demonstrating that the *in vivo* environment is beneficial for advanced neuronal maturation compared to neurons kept *in vitro*. Neurons from transplanted organoids formed functional synaptic connections with rat thalamic and cortical circuits, responded to sensory input, and extended axonal projections into the rat brain that influenced rat behavior. Clear differences in neuronal morphology and electrical activity were observed for transplanted organoids derived from patients with Timothy syndrome, a rare genetic disorder linked to autism and epilepsy, compared to isogenic controls, underscoring that this approach is promising for disease modeling.

In addition to the challenges of slow development, high variability, low throughput, and limited network maturation, human brain organoids recently also raise ethical concerns (Kataoka et al., 2025). As these models become increasingly complex, exhibiting features such as spontaneous electrical activity and primitive sensory structures, questions are raised about their potential for consciousness or sentience. While current human brain organoids remain far from achieving such capabilities, these concerns highlight the need for ongoing ethical oversight and clear guidelines as the field advances.

### Mouse brain organoids

The application of human brain organoids is constrained by high cost, lengthy differentiation times, and substantial variability between batches. Mouse brain organoids (Lloyd-Davies Sánchez et al., 2024), derived from either mouse ESCs or iPSCs, address these limitations and leverage the extensive knowledge derived from the most widely used mammalian *in vivo* model in neuroscience. The extensive molecular (Langlieb et al., 2023), physiological (Demin et al., 2021), and developmental data from the mouse brain provide a valuable reference framework for benchmarking and validating these emerging *in vitro* systems. Such data help identifying potential artefacts and remaining biological limitations when comparing organoid models to the *in vivo* biology they aim to represent. Moreover, the generation of mouse organoids, in combination with human brain organoids, offers a platform that can help bridge findings between traditional *in vivo* studies and human clinical trials, improving translational reliability. Several established protocols to generate mouse brain organoids already exist, including cerebellar (Guglielmi et al., 2024), hippocampus (Ciarpella et al., 2023) and forebrain models (Medina-Cano et al., 2022), recapitulating aspects of cortical layering, synapse formation, and neuronal activity *in vitro*.

While mouse brain organoids offer strategic advantages, their use is not without challenges. The generation process remains relatively long (several weeks) and costly, and these models still lack the full structural and functional complexity of the intact brain, such as vascularization, long-range connectivity, and full neuronal maturation (Medina-Cano et al., 2025). Moreover, because mouse organoids model murine rather than human neurobiology, their relevance to human biology is limited, which significantly constrains the interpretation and extrapolation of disease mechanisms compared with human-derived *in vitro* models. While mouse organoids are valuable for studying fundamental aspects of neural development, regional specification, and circuit formation, their ability to recapitulate disease-specific phenotypes such as neurodegeneration, synaptic dysfunction, or network hyperexcitability remains limited. The gap underscores the need for genetically tractable, reproducible mouse organoid systems that more accurately reflect pathological processes. Thus, although they provide a valuable mammalian system to refine and complement existing models, further methodological optimization and validation are required before they can fully capture the complexities of neural development and disease (Marshall and Mason, 2019).

### Zebrafish model

**Figure 2:**
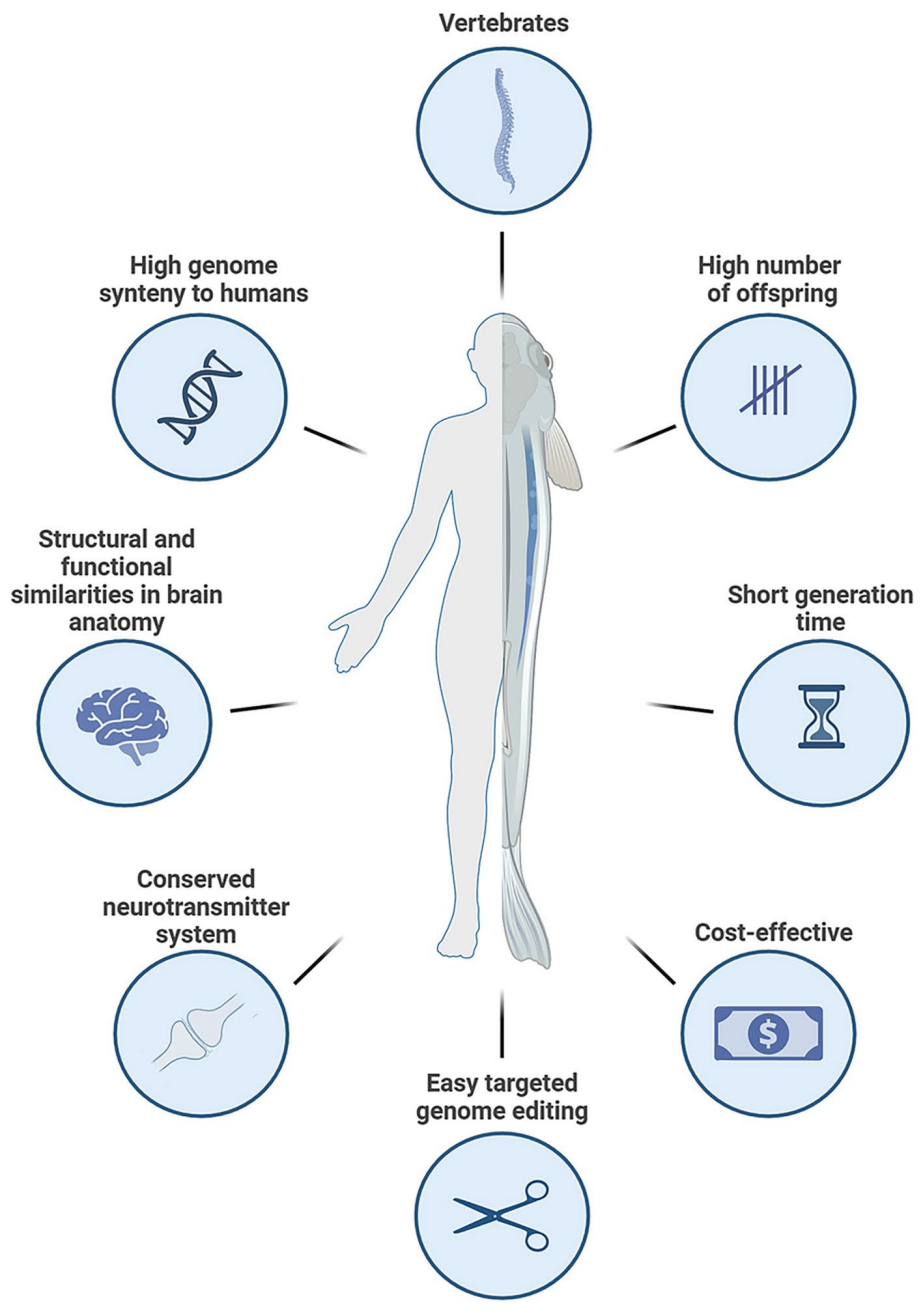
Advantages and relevance of zebrafish as a vertebrate model in neuroscience research. Zebrafish offer high level of conservation with regards to the human reference genome, conserved brain structure and neurotransmitter systems, ease of genome editing, rapid reproduction, short generation time, and cost-effectiveness, making them ideal for neurodevelopment and toxicity studies, functional genomics, disease modeling, and drug discovery. Figure created with Biorender.com.

Zebrafish (*Danio rerio*) are a non-mammalian vertebrate species with several unique advantages that make them particularly well-suited as *in vivo* NAMs when used at embryonic and early larval stages (Figure 2). Zebrafish embryos are not legally classified as protected animals until 120 h post-fertilization (hpf) when they are independently-feeding, aligning their use with international regulatory and ethical considerations, such as the EU Directive 2010/63/EU on the protection of animals used for scientific purposes (EU, 2010). This makes them a refined alternative that significantly reduces the need for higher-order vertebrates while maintaining biological relevance and *in vivo* complexity. This aspect makes zebrafish a non-animal *in vivo* model during early development that is particularly attractive for early-stage research and drug discovery and development, especially in toxicological assessments and pharmacological studies where large-scale screening is essential. As such, zebrafish bridge the gap between traditional *in vitro* assays and mammalian *in vivo* models, effectively fulfilling the 3Rs principles (Strahle et al., 2012; Cassar et al., 2020).

Zebrafish have a high level of conservation with regards to the human reference genome, with approximately 70% of human genes having at least one zebrafish ortholog, and up to 84% of genes known to be associated with human disease having a zebrafish counterpart (Howe et al., 2013). Despite being small and evolutionarily distant, zebrafish exhibit remarkable conservation in vertebrate brain regions, gene expression patterns (Mueller and Wullimann, 2009) and neurotransmitter systems (Guo, 2009), making them a valuable model for studying the CNS, neurological diseases, and drug effects. However, they lack the highly complex cortical architecture seen in humans and other mammals, not possessing layers and gyri (Mueller and Wullimann, 2003). Importantly, zebrafish embryos are transparent and develop rapidly *ex utero*, allowing real-time observation of (neuro)developmental processes and organ formation within just a few days post-fertilization. Moreover, embryos and larvae can be arrayed in microtiter plates (body length ≤4.5 mm). This makes them ideal for high-throughput screening in drug discovery and toxicology, where hundreds of compounds are tested quickly and cost-effectively in multi-well plate formats. Their susceptibility to genetic manipulation (e.g., CRISPR/Cas9) further enhances their utility in modeling human diseases and understanding gene function (Chang et al., 2013; Hwang et al., 2013).

Thanks to these strengths, zebrafish are well-accepted as a reliable, rapid, inexpensive, and medium- to high-throughput *in vivo* model in toxicological studies (Rosa et al., 2022; Zhao et al., 2024; Bult and Sternberg, 2023; Yamamoto et al., 2024), drug discovery screening, and (patho)mechanistic investigations (Bambino and Chu, 2017; Shao et al., 2024; Tang et al., 2023). For example, the Zebrafish Embryotoxicity Test (ZET) is a widely used *in vivo* assay for evaluating the developmental toxicity and teratogenicity of chemicals (Achenbach et al., 2020; Brannen et al., 2010; Gustafson et al., 2012). It can detect effects of known human teratogens such as thalidomide and valproic acid (Hoffmann et al., 2021), by monitoring morphological changes during early embryonic development. The ZET is included in integrated testing strategies (ITS) for developmental and reproductive toxicity (DART) testing (Burbank et al., 2023), alongside *in silico* models from the OECD QSAR (Quantitative Structure–Activity Relationships) Toolbox (Lavado et al., 2020; Lovric et al., 2021; Saavedra and Duchowicz, 2021) and *in vitro* assays like the human iPSC-based devTox assay (Zurlinden et al., 2020), to support comprehensive chemical risk assessment. Zebrafish are also valuable in predicting human developmental toxicity (Boyd et al., 2016; Padilla et al., 2012), as evidenced by data from the US Environmental Protection Agency (EPA) “ToxCast” program (launched in 2007) (Dix et al., 2007), and comparative studies further state that zebrafish can serve as alternatives to traditional mammalian models for developmental toxicology (Nishimura et al., 2016; Kily et al., 2008; Wester et al., 2004). In the field of neurotoxicity, zebrafish are widely used to detect toxicant-induced functional changes in the nervous systems, including behavioral alterations (e.g., changes in locomotor activity) and electrophysiological disturbances (e.g., seizure-like discharges) (Fitzgerald et al., 2021; Tomasello and Wlodkowic, 2022; Forner-Piquer et al., 2021; Chai et al., 2024). Moreover, zebrafish are a state-of-the-art model for studying neurological disorders such as Alzheimer’s disease, Parkinson’s disease and epilepsy. Standard read-outs include molecular, behavioral and electrophysiological measurements. Both chemically-induced and genetic zebrafish models significantly contributed to the discovery of therapeutic compounds for neurological conditions. A notable example is the zebrafish model of Dravet syndrome (Griffin et al., 2021), a severe drug-resistant childhood epilepsy caused by mutations in the *SCN1A* gene. This model has been instrumental in identifying repurposed drug candidates such as clemizole (Baraban et al., 2013) using high-throughput screening and in gaining mechanistic insights in fenfluramine (Sourbron et al., 2017), a recently approved therapeutic agent. In addition, the zebrafish ethyl ketopentenoate (EKP) model of drug-resistant seizures was used for identifying clioquinol as a repurposed drug candidate, which is now in clinical trials for a new disease indication (Thevissen et al., 2025). Beyond drug discovery, zebrafish disease models are invaluable for (patho)mechanistic research, offering insight into pathological processes that in turn can result in the discovery of novel drug targets and the development of innovative therapies. For example, a study in a zebrafish model of DS revealed novel early mechanisms of disease pathogenesis, describing dynamic changes in neuronal and glial cell populations during epileptogenesis, and provided first-time evidence for potential disease modification by fenfluramine (Tiraboschi et al., 2020). Moreover, in zebrafish models of Alzheimer’s disease, a pathological loop linking oxidative stress, neuroinflammation, and gut microbiota dysbiosis along the gut-brain axis has been demonstrated (Qu et al., 2023; He et al., 2024). Finally, zebrafish are invaluable for functional genomics, providing a versatile *in vivo* system to investigate how genes and genetic variants contribute to biological processes and human disease (Howe et al., 2013; Dore et al., 2025; Patton et al., 2021). Many genetic mutant lines and morphant models have been generated, enabling, among others, the efficient validation of candidate disease genes identified by human GWAS or sequencing studies, as well as the investigation of how genetic mutations affect behavior and neurobiology. This is illustrated by recent reports employing high-throughput generation and characterization of genetic mutants, demonstrating the capacity of zebrafish for rapid disease modeling across conditions ranging from epilepsy (Griffin et al., 2021) to other complex neurological traits (Kroll et al., 2021).

### Zebrafish brain organoids

Traditional human brain organoids mimic key features of the developing human brain and model neurological diseases, but they are limited by high cost, long culture times, variability and lack of direct comparison to an *in vivo* reference. Mouse organoids offer a faster, more cost-effective, and ethically simpler model than human organoids and mice themselves (Marshall and Mason, 2019) and are used mainly for developmental research. Although they show potential for disease modeling and drug testing, these applications remain largely experimental (Marshall and Mason, 2019). They also remain relatively costly, slow to develop, and less amenable to high-throughput studies. In contrast, zebrafish embryos develop rapidly, are cost-effective and are highly accessible for *in vivo* studies, but after 120 hpf they are considered animal models, which introduces regulatory and ethical constraints. Noteworthy, brain structures such as the BBB are still developing beyond this stage (Kimmel et al., 1995), which can necessitate the use of older larvae and hence animal studies. To bridge these gaps, zebrafish brain organoids provide a pioneering complementary model that combines the strengths of *in vitro* organoid technology with those of the zebrafish, including rapid development, offering a novel and high-throughput platform for drug discovery and development and for studying neural development and neurotoxicity in a controlled yet physiologically relevant and ethically favorable context.

#### 2D cultures of primary fish-derived embryonic stem cells

In the last decades, several studies (Ho et al., 2014; Hong et al., 1996; Robles et al., 2011; Yi et al., 2010) reported attempts to culture embryonic stem (ES)-like cells from fish blastula-derived cells, but protocols vary between laboratories and maintaining true ES cell cultures from zebrafish is technically demanding (He et al., 2006; Collodi et al., 1992; Ma et al., 2001; Xing et al., 2008; Driever and Rangini, 1993). Additionally, gene-targeting approaches have been applied in these species to facilitate the development and characterization of such ES-like cell lines (Hong et al., 1996). The established ES-like cell lines from zebrafish support the notion that pluripotency is conserved among vertebrates (Collodi et al., 1992; Sun et al., 1995), but cultures show heterogeneity, and spontaneous differentiation is a challenge and contributes to the limited number of stem cell lines available (Goswami et al., 2022). Their ability to differentiate and self-organize into polarized aggregates have been demonstrated by specifying all three germ layers (i.e., ectoderm, mesoderm, and endoderm) (Fulton et al., 2020; Schauer et al., 2020), thereby recapitulating key aspects of early embryonic patterning and morphogenesis. These structures exhibit a strong resemblance to mammalian ES cell-derived gastruloids (Beccari et al., 2018; van den Brink and van Oudenaarden, 2021; Moris et al., 2020; Turner et al., 2017). Moreover, zebrafish primary ES cells have already been differentiated into functional cardiomyocytes exhibiting contractile kinetics and electrophysiological features (Xiao et al., 2016). Several studies demonstrated that ES cells derived from zebrafish can differentiate into both neurons and astrocytes *in vitro* (Ghosh et al., 1997), and primary cultures of zebrafish neural cells have been established (Patel et al., 2019). However, they typically have low survival rates *in vitro*, which poses challenges for long-term culture and functional studies.

#### 3D fish brain organoids

Reports of zebrafish-derived tissue or dissociated cells forming complex, organized neural structures similar to those seen in mammals are limited. Consequently, and due to the interest in human models, the organoid field largely remained restricted to mammalian species (i.e., rodents and humans). However, Zilova et al. (2021) demonstrated the successful derivation of brain organoids from rapidly developing teleosts (i.e., zebrafish and medaka), marking an important advance in this area. This study shows that primary embryonic pluripotent cells derived from blastula-stage embryos of medaka and zebrafish can form neuroepithelial aggregates that develop into anterior neuronal organoids expressing the general neuronal marker Sox2 (Pevny and Placzek, 2005; Wegner and Stolt, 2005) and the forebrain marker *Otx2* (Acampora et al., 1995; Simeone et al., 1993; Tian et al., 2002). Furthermore, expression of HuC/D, a marker of early post-mitotic neurons (Good, 1995; Kim et al., 1996; Park et al., 2000), indicates that the fish organoids show onset of neuronal differentiation. Remarkably, within just four days, these fish-derived cells undergo efficient brain differentiation and morphogenesis in culture demonstrating that brain region formation in these organoids mimics the *in vivo* cell migration patterns.

**Figure 3:**
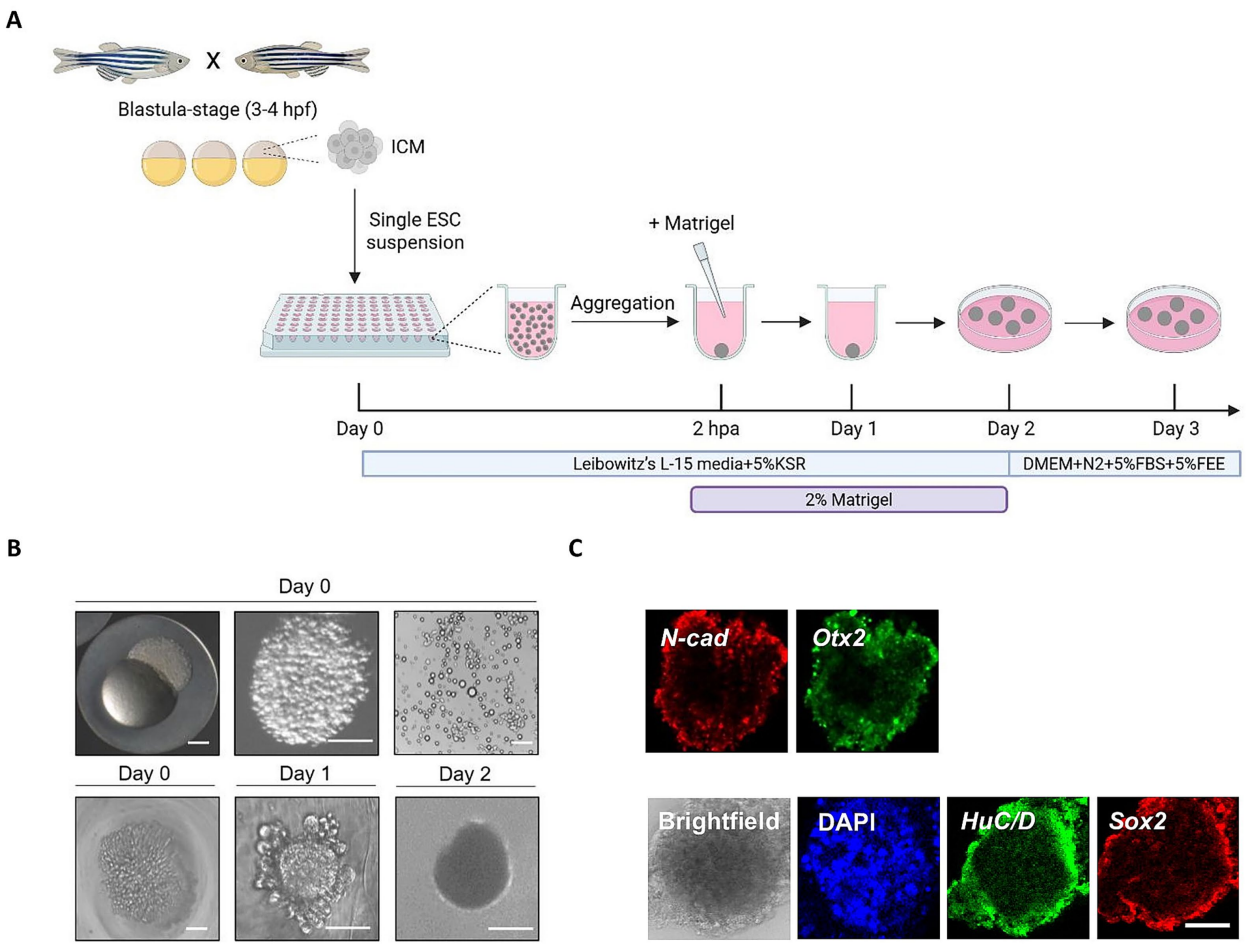
Generation of zebrafish primary pluripotent cell-derived aggregates. **(A)** Schematic representation of aggregate generation, its timeline and culture conditions. The protocol for blastula-stage zebrafish embryo dissociation and 3D aggregate culture was reproduced from Zilova et al. (2021) with minor modifications. The aggregates were incubated at 26 °C in the incubator without CO_2_ control and Matrigel was added at 2 h post-aggregation (hpa) to a final concentration of 2%. From day 1 onward, aggregates were incubated at 26 °C and because CO_2_ control was not possible, media was supplemented with 20 mM HEPES, pH = 7.4. At day 2, the aggregates were transferred to a low binding culture plate and maintained in 3D suspension culture conditions. The gross morphology of the aggregates was analyzed at days 1 and 2. KSR, knockout serum replacement; FBS, fetal bovine serum; FEE, fish embryonic extract. **(B)** Dark-field images of a blastula-stage embryo, a blastula-derived intact inner cell mass after cutting, blastula-derived cell suspension, re-aggregated cells and the gross morphology of aggregates at days 1 and 2 after re-aggregation. **(C)** Optical sectioning showing aggregate organization at day 2 visualized by immunostaining against the neuroepithelium-specific marker *N-cadherin* (*N-cad*), forebrain anterior neuronal marker *Otx2*, marker of early post-mitotic neurons *HuC/D*, general neuronal marker *Sox2*, and co-stained with DAPI nuclear stain. Whole-mount staining was performed as described by Zilova et al. (2021) using the same antibodies. Scale bars: 100 μm. Fixed organoid samples were imaged with Leica Sp8 confocal microscope. Gross morphology of organoids was assessed by the EVOS Floid imaging system.

We reproduced their published protocol, with minor modifications (Figure 3A), confirming its reproducibility and the feasibility to generate comparable neuroepithelial structures. Our observations confirm that primary zebrafish-derived pluripotent ES cells form neuroepithelial aggregates and reproducibly differentiate into brain structures within just a few days (Figures 3B,C). Notably, while zebrafish can produce several hundred embryos per spawning (i.e., 100–300 viable embryos under optimal conditions), the manual dissection and isolation of ESCs from each embryo is labor-intensive and time-consuming. Thus, large-scale production of zebrafish organoids currently demands significant time and labor input. The fish-derived organoids are generated from aggregates of fewer than 1,000 embryonic stem cells, which is roughly half the number of cells in a single blastula-stage embryo (Kimmel et al., 1995). This means that up to three organoids can be produced from just one embryo, reducing the total number of embryos needed and supporting the 3Rs principle in animal research. Re-aggregation of dissociated primary pluripotent cells from blastula-stage zebrafish embryos is rapid (approximately 2–3 h) and highly efficient (~100%), resulting in a smooth and compacted morphology of aggregates by day 1. From day 2 onward, cells in the peripheral layer expressed neuroepithelial markers such as *N-cadherin*, confirming the acquisition of neuroepithelial identity. The aggregates also expressed the general neuronal marker *Sox2* and the forebrain marker *Otx2*, indicating that the tissue adopted an anterior neuronal fate. This differentiation protocol leverages the assembly of cells into various organ-like structures, highlighting how *in vitro* systems provide a unique opportunity to study the fundamental principles of organ formation.

Moreover, Zilova and colleagues showed that genetic factors critically influence morphological changes that mirror the *in vivo* situation. The high efficiency and rapid development of fish-derived organoids, combined with advanced genome editing (CRISPR/Cas9), enabled them to examine the coordinated expression of key eye field transcription factors, such as *Rx3* (Chuang et al., 1999; Loosli et al., 2003), in the anterior neural plate. Their data show that fish-derived organoids form retinal neuroepithelium with multiple optic vesicles under the control of *Rx3*, with the onset of retinal fate being genetically timed and strictly dependent on expression of this transcription factor. Intriguingly, in a fraction of organoids, retinal and non-retinal domains showed an arrangement, resembling a simple “head with two eyes.” This demonstrates that key aspects of organogenesis, early eye development in this case, are faithfully recapitulated *in vitro* in a dish maintaining the same genetic and developmental programs compared to the embryo. Moreover, compared to their human and murine counterparts, zebrafish organoids develop much faster, progressing about ten times faster than murine models (Kuwahara et al., 2015; Nakano et al., 2012; Eiraku and Sasai, 2011) and closely following the *in vivo* developmental timeline, which reflects the species-specific differences in developmental speed.

##### Application potential

Compared to conventional *in vivo* mouse and zebrafish experiments, brain organoids offer a simplified, controllable platform that reduces ethical and logistical barriers while still capturing key aspects of vertebrate neurodevelopment. For human brain organoids, numerous standardized protocols are established, supporting a wide range of applications, including patient-specific disease modeling and neurotoxicity research. Mouse brain organoids similarly recapitulate many aspects of early neurodevelopment and have been successfully generated using several published protocols. However, their use is limited to fundamental neurodevelopmental investigations, with disease modeling applications being less established. The simple and reproducible methodology for generating zebrafish brain organoids makes this approach attractive to a wide range of research groups, both to those experienced in pluripotent stem cell culture, but lacking zebrafish expertise or aquatic facilities, and to labs working with fish models but with limited stem cell experience. By providing an accessible and consistent way to produce organized neural tissue *in vitro*, this protocol broadens the use of zebrafish as a model system beyond traditional whole-animal studies.

**Figure 4:**
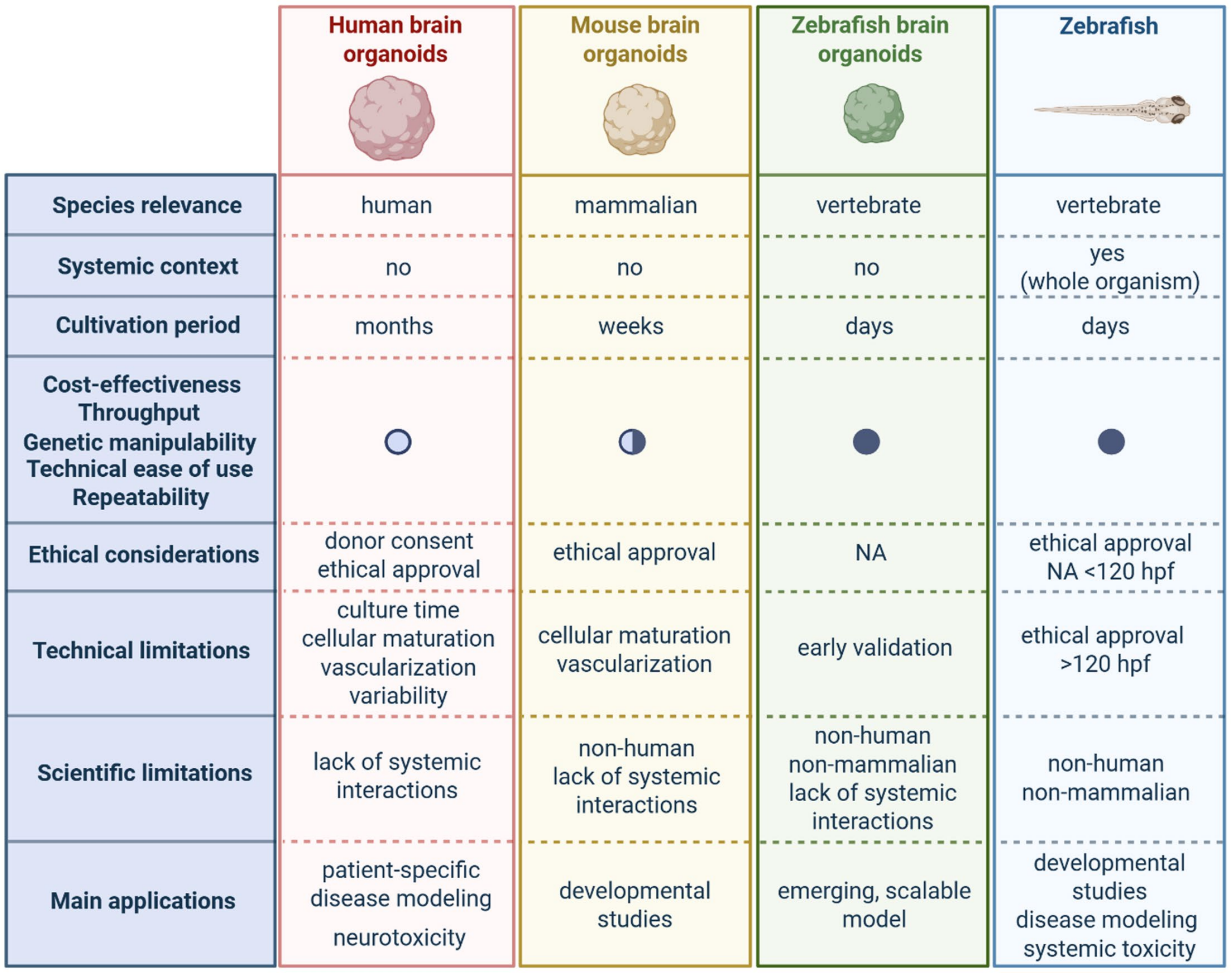
Comparative overview of key features, strengths, and limitations of selected New Approach Methodologies (NAMs) in neuroscience. Comparison of human, mouse and zebrafish brain organoids, and zebrafish models, illustrating differences in species relevance, systemic context, and time to produce. Differences in cost-effectiveness, throughput, genetic manipulability, technical ease of use and repeatability are indicated by circles: filled circle, high; semi-filled, intermediate; open circle, low. Ethical considerations, main limitations, and main applications are also listed. NA, not applicable; hpf, hours post-fertilization. Figure created with Biorender.com.

However, like all NAMs, brain organoids face practical and technical limitations (Figure 4). One of the major limitations of current brain organoids (human, mouse and fish) is that they lack vascularization leading to cell death and limited growth (Qian et al., 2019). Moreover, because stable ESC lines are not (yet) available for zebrafish, in contrast to mouse and human, organoid generation requires the use of zebrafish embryos, limiting yield to only a few organoids per embryo, restricting scalability and reproducibility of zebrafish brain organoids. While optimizing automation of blastula dissection and organoid aggregation could reduce variability and labor demands, establishing stable fish stem cell lines would be a pivotal step towards improving standardization. This would also support broader implementation across laboratories. Potentially, the number of organoids produced from just one embryo could be increased when the earlier stage totipotent blastomeres would be considered. Regardless, the availability of ES cells in culture would be most optimal. Mouse organoids (Hu et al., 2018; Lee et al., 2020; Taguchi and Nishinakamura, 2017), on the other hand, are generated from ESC or iPSC stable lines, eliminating the need to isolate ESCs from embryos for each differentiation. Therefore, they have no impact on the number of embryos used.

Although mice have a cerebral cortex, it is smaller and less complex than the human cortex. Organoids reproduce these species-specific differences with human organoids showing longer progenitor cycles and abundant outer radial glia (Li et al., 2017), while mouse organoids reflect the faster, simpler murine developmental program, making them valuable for studying brain development *in vitro* as mouse brain organoids are grown in a shorter time period than human ones (weeks versus months). Moreover, mouse brain organoids are widely used to compare phenotypes between *in vitro* mutant organoids and *in vivo* embryos (Lloyd-Davies Sánchez et al., 2024), generate region-specific organoids (Medina-Cano et al., 2025; Medina-Cano et al., 2022), and improve organoid models by developing and optimizing new technologies. The current fish organoid model develops even faster (days) and demonstrates the capacity of zebrafish pluripotent stem cells to self-organize into complex neural tissues. Although it is a limitation that cortical-like structures cannot be reproduced in zebrafish organoids due to the distinct forebrain architecture of zebrafish, the zebrafish pallium and subpallium are considered the functional and molecular analogs of the mammalian cortex and basal ganglia (Pang et al., 2025). Consequently, approaches could be optimized to generate organoids that model zebrafish-specific brain regions, such as the telencephalon, thereby capturing the diversity of neuronal and glial cell types and providing a faster platform to explore conserved principles of vertebrate forebrain development in a more simple model.

Furthermore, the differentiation protocol, initially designed to generate anterior neural and retinal fish organoids, holds great promise for studying a variety of other organs beyond the brain. Fish pluripotent stem cells have the capacity to differentiate into multiple tissue lineages, making them a versatile platform for modeling organ development *in vitro*. By adjusting culture conditions, signaling cues, and extracellular matrix components, this approach can be adapted to produce organoids representing kidney, heart, liver, gut, and other fish tissue, providing valuable insights into vertebrate organogenesis. This hence is expected to mirror the versatility observed in mouse organoid systems, where similar *in vitro* approaches have successfully produced kidney (Taguchi and Nishinakamura, 2017), liver (Hu et al., 2018), and heart organoids (Lee et al., 2020), highlighting the broader applicability of organoid technology for modeling diverse vertebrate tissues.

Beyond serving as a platform for *in vitro* organogenesis studies, as demonstrated by Zilova et al., the novel fish brain organoid model provides a scalable, cost-effective system for disease modeling, functional genomics, and high-throughput drug screening. For example, while studies have shown that human brain organoids combined with CRISPR/Cas9 gene editing (Chen et al., 2021) successfully model genetic epilepsies (De Meulemeester et al., 2024), revealing phenotypes such as neuronal hyperexcitability and disrupted network synchrony, this approach is associated with a high cost, technical complexity, and slow maturation. Zebrafish brain organoids could help address these constraints by offering a complementary model that bridges the gap between the rapid, whole-organism advantages of zebrafish larvae and organoid technology. Together, these complementary models cover a range of applications in neuroscience, including zebrafish brain organoids for scalable mechanistic and screening applications, mouse brain organoids for neurodevelopmental studies, human brain organoids for patient-specific disease modeling and translational relevance, and *in vivo* vertebrates animal models (zebrafish, mouse, etc.) for disease modeling and systemic, behavioral and neurotoxicity studies.

In recent years, numerous studies have been published using cerebral organoids, with the vast majority employing human PSCs. This predominance is due to the key strength of human brain organoids that they can be derived from patient-specific pluripotent stem cells, capturing an individual’s complete genetic background (Takahashi and Yamanaka, 2006). This is especially important for complex polygenic disorders, such as epilepsy, Alzheimer’s and Parkinson’s disease, where disease penetrance is highly influenced by an individual’s genetic background (Lancaster and Knoblich, 2014). The latter represents one of the main limitations of organoids derived from alternative organisms, such as fish and mice, which cannot fully capture human-specific genetic variability and disease mechanisms. However, by combining mouse and zebrafish brain organoids with advanced gene editing tools, researchers can perform targeted functional genomics studies to dissect genotype–phenotype relationships, identify novel genetic drivers of neurological disorders, and evaluate gene-targeted therapies prior to validation studies in human brain organoids. Many zebrafish models of genetic epilepsies already exist (Copmans et al., 2017), such as the larval zebrafish *scn1lab^−/−^* mutant model of Dravet syndrome, and pluripotent cells derived from these embryos can be used to generate disease-specific brain organoids. These fish organoids would be particularly well suited for high-throughput drug screening, enabling fast and efficient assessment of drug efficacy and safety in a non-animal vertebrate context. Promising conditions identified in these screens, such as active compounds and safe concentrations, could then be tested in human brain organoids, providing a rapid pre-screening step before more resource-intensive human studies. Notably, because stable, long-term zebrafish ESC lines are not (yet) available, genome editing for zebrafish organoids must be carried out in embryos, necessitating embryonic screening, ESC isolation and subsequent organoid generation, which increases embryo use. In contrast, mouse ESCs and iPSCs are maintained as stable lines, allowing genome editing entirely *in vitro*, facilitating controlled studies of genetic variants in a mammalian context. While zebrafish organoids provide speed and high-throughput potential, mouse organoids provide a genetically tractable system with closer physiological relevance to humans, allowing a strategic combination of both platforms for preclinical research (Figure 4). Hence, complementing the insights gained from both whole-animal and human *in vitro* models.

In addition to disease modeling and drug discovery, mouse and zebrafish brain organoids also hold significant potential as an *in vitro* platform for neurotoxicity and ecotoxicity screening of pharmaceuticals, chemicals and environmental toxicants. Zebrafish already demonstrated strong relevance for studying ecological neurotoxicology, illustrating the interconnected pathways through which environmental pollutants impair brain health, including gut-brain axis dysregulation (Sun et al., 2025; Xu et al., 2025). In a recent study (Leekitratanapisan et al., 2025), zebrafish were used to reveal behavioral effects of complex wastewater micropollutant mixtures, further highlighting their value as a powerful vertebrate system for mechanistic and ecological assessments of environmental contaminants. These findings underscore the potential of zebrafish brain organoids as an innovative model to extend such investigations *in vitro*, enabling the study of neurotoxic mechanisms, dose–response relationships, and systemic neuroinflammatory pathways under controlled yet environmentally relevant exposure conditions. Notably, this emerging model aligns with the EU’s One Health approach by offering a safe, sustainable and animal-reducing alternative that addresses health risks at the human-animal-environment interface. To demonstrate their feasibility for safety and toxicity testing, however, it is crucial to systematically assess their morphological, transcriptional, and functional changes in response to test compounds. Such assessments should include for example the detection of dose-dependent toxicity, off-target effects, and developmental neurotoxicity, ensuring that these organoids show high predictive validity and reliably reflect the impact of toxic agents on brain structure and function, while providing an efficient complement to existing vertebrate and human-based NAMs.

### Model validation

The importance of integrated, multi-level validation of newer organoid systems cannot be overstated to ensure their applicability. Both mouse and zebrafish brain organoids require morphological, functional, and molecular characterization, benchmarked against human brain organoids, whole mouse (brain), or whole zebrafish (brain), depending on the species. Such validation ensures that organoids are biologically faithful and translationally relevant by confirming that they accurately recapitulate *in vivo* neurodevelopmental processes and disease mechanisms. The characterization and demonstration of their construct (pathomechanisms), face (clinical symptoms) and predictive (treatment) validities will support their use in mechanistic studies, disease modeling, and high-throughput drug discovery.

Human brain organoids have set the precedent for validation of *in vitro* models (Lloyd-Davies Sánchez et al., 2024). Morphological validation includes analysis of organoid size, shape, cellular composition, and structural organization using immunohistochemistry and imaging techniques. Notably, the size, shape and viability of human brain organoids can vary significantly both within and between batches. To address this heterogeneity, a level of standardization needs to be achieved to benchmark selected protocols which can be applied to newly adopted differentiation protocols to assure the consistency of data across experiments. Researchers have focused on high-throughput imaging (Handcock et al., 2025; Deininger et al., 2023), 3D spatial single cell and proteomic-based analysis methods (Albanese et al., 2020) to assess organoid morphology. However, investigating the cellular architecture and diversity of human and mouse brain organoids typically requires sectioning prior to immunohistochemistry (IHC) (Bremond Martin et al., 2021) due to their large tissue sizes (typically thicker than 1 mm) (Pasca et al., 2015). In contrast, zebrafish brain organoids are small enough to perform whole-mount staining, eliminating the need for sectioning. However, the limited availability of validated antibodies for zebrafish tissues remains a challenge for detailed cellular characterization.

In addition, numerous studies characterized the transcriptomic (Cheroni et al., 2022; Tanaka et al., 2020) and proteomic (Elmkvist et al., 2024) profiles of human and mouse brain organoids during neuronal development to assess cellular identity, developmental dynamics, and cytoarchitecture, demonstrating that organoids largely recapitulate *in vivo* programs with only minor differences (Bhaduri et al., 2020). This high degree of concordance between *in vitro* and *in vivo* developmental trajectories is not limited to mammalian systems. Zebrafish brain organoids also exhibit similar fidelity as shown by Zilova et al. (2021) as pluripotent zebrafish embryonic cells self-organize into retinal tissue that mirrors early *in vivo* eye development. This further underscore the broader applicability of organoid systems across species for modeling conserved neurodevelopmental programs.

The electrical properties of single neurons *in vitro* are measured using whole-cell patch-clamp recordings, which is the gold standard in electrophysiology today. This technique enables detailed characterization of intrinsic neuronal properties, including spontaneous fluctuations in membrane potential, action potential firing, and synaptic activity. Due to their small size, zebrafish brain organoids can be patched intact (Landry et al., 2023), in contrast to larger human- and mouse brain organoids, which require slicing to access individual cells for recording (Segev et al., 2016). This offers a technical advantage for high-resolution functional studies. Multi-electrode arrays (MEAs) enable non-invasive recording of spontaneous or evoked electrical activity across networks of neurons within the organoid. This technique measures spiking rates, burst patterns, and network synchrony, reflecting functional connectivity and coordinated circuit behavior (Sit et al., 2024). The observed electrophysiological phenotypes of mouse and zebrafish brain organoids should be compared to neurons *in vivo*, in human brain organoid models *in vitro*, and even to patient-derived post-mortem brain tissue samples as a clinical reference, to assess successful recapitulation. Furthermore, if these models reliably reproduce treatment responses seen *in vivo* and in patients, this will provide predictive validity and the potential to complement human brain organoids and whole-animal studies for disease modeling and high-throughput drug screening.

### Future integration of emerging NAMs in neuroscience

Within the growing landscape of NAMs in neuroscience, brain organoids are promising *in vitro* tools among others. Although the methodology for generating these organoids is optimized to ensure reproducibility, comprehensive functional validation remains essential to establish their reliability and relevance. Compared to mammalian brain organoids (human and mouse), zebrafish brain organoids offer strategic advantages, including lower cost, faster production, great scalability, and simpler protocols, making them an attractive addition to the NAMs toolkit for early discovery purposes. As novel, early-stage NAMs, both mouse and zebrafish brain organoids hold significant potential for preclinical research, yet further research is essential to rigorously assess their predictive power. To reach their complete potential, clear standards, refinements in methodology, and systematic incorporation into preclinical testing frameworks are needed. Emphasizing reproducibility, harmonization, and cross-system evaluation can help advance brain organoids toward a more predictive and ethically responsible neuroscience paradigm.

Moving forward, the scientific roadmap for brain organoids, among other NAMs, should focus on three complementary action paths that together enable their systematic validation, regulatory alignment, and integration into multi-model research pipelines. First, cross-laboratory reproducibility and shared reference datasets are essential. Coordinated inter-laboratory trials, with academic and regulatory partners such as the OECD and EURL ECVAM, should assess reproducibility of key morphological, transcriptomic, and electrophysiological endpoints. Developing openly accessible reference datasets and standardized benchmark compounds and neurotoxicants will support comparison of *in vitro* results with *in vivo* data. Consistency will further depend on quantifiable quality metrics such as organoid size distribution, cell-type composition, viability, and neural activity.

Second, alignment with OECD and regulatory validation frameworks will be critical to promote broader acceptance of available and emerging NAMs. Regulatory acceptance requires demonstrating equivalence or superiority to existing *in vitro* and *in vivo* methods. Endpoints should be mapped to existing OECD test guidelines for developmental neurotoxicity and incorporated into the OECD’s modular validation approach. Defining performance standards, including accuracy, precision, and biological relevance relative to established zebrafish and rodent data, will further support transparent regulatory pathways and harmonized implementation.

Third, integration of complementary models will strengthen translational value and predictive power. Developing mouse and zebrafish brain organoids should not aim to replace but to complement and accelerate existing human *in vitro* and conventional *in vivo* animal approaches. Simple 2D cultures are useful for mechanistic studies and screening, non-human brain organoids offer an intermediate platform, with zebrafish brain organoids as an emerging and scalable model for disease modeling and toxicity testing, and advanced human brain organoids provide patient-specific insights and translational relevance. Zebrafish embryos and larvae add the complexity of a whole organism, enabling behavioral and neurophysiological studies (high face validity) as a pre-rodent model and NAM. Used together, these models can address the limitations of any single system and create a more robust, ethically responsible pipeline for preclinical research. Parallel use of the non-mammalian zebrafish brain organoids and mammalian mouse and human organoids further enable cross-species comparisons, helping to identify conserved mechanisms of neurodevelopment. Computational modeling and quantitative adverse outcome pathways can further connect cellular perturbations observed in organoids with organismal phenotypes, improving mechanistic interpretation and supporting extrapolation to human health outcomes.

**Figure 5:**
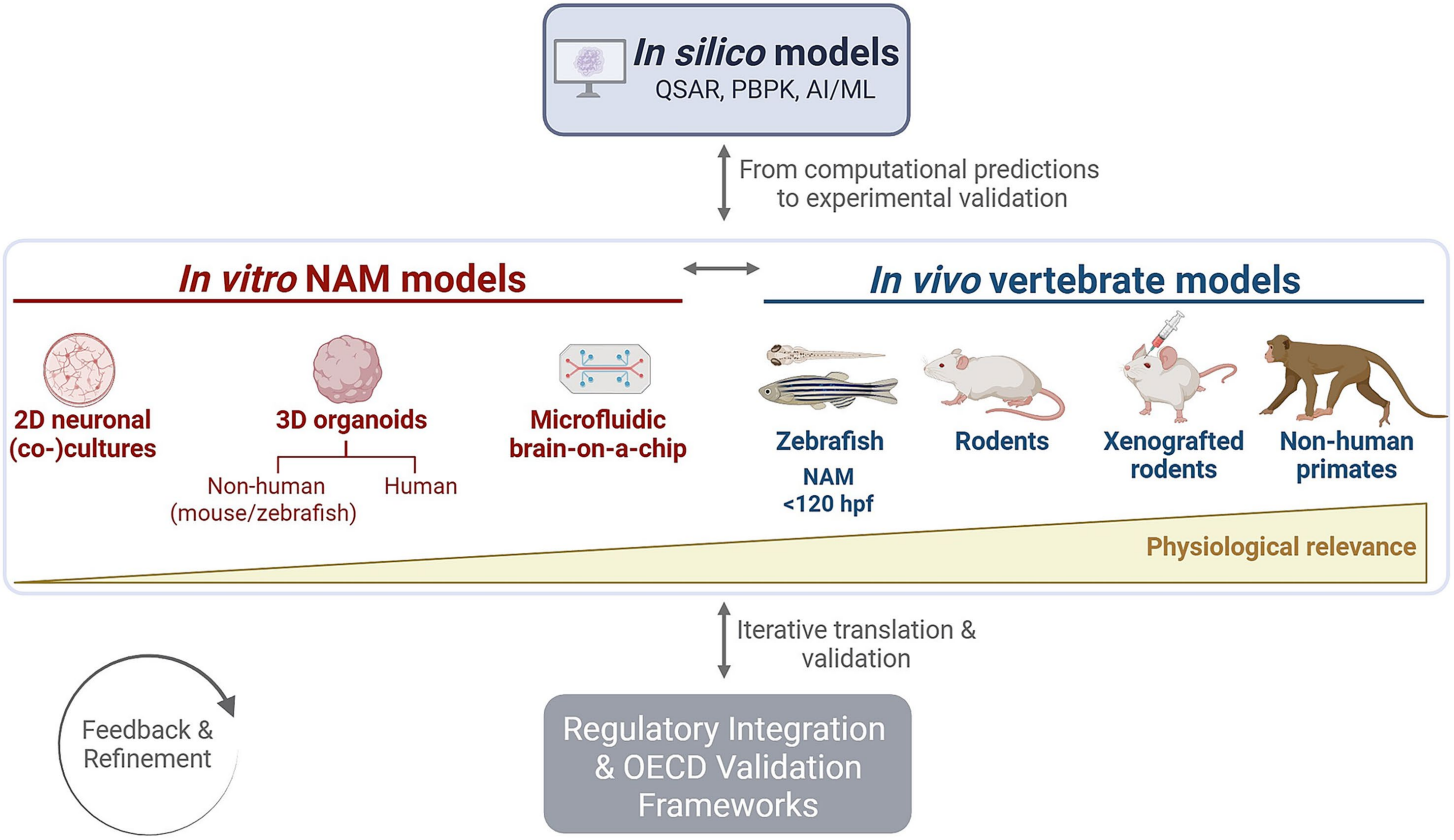
Integration of new approach methodologies (NAMs) across the translational spectrum. This multi-level flowchart illustrates the iterative and modular integration of NAMs, linking *in silico, in vitro,* and *in vivo* approaches through continuous feedback. *In silico* models generate computational predictions that guide experimental design. *In vitro* assays employ increasingly complex models, from simple 2D cultures for mechanistic studies to 3D organoids, including mouse and zebrafish organoids as intermediate systems, and ultimately human organoids for advanced validation. Depending on the research question, one or more *in vitro* or *in vivo* models may be selected, typically, but not necessarily, progressing from simpler to more physiologically relevant systems. *In vivo* data can further refine and validate predictions, reinforcing the iterative NAMs framework. Finally, integration into regulatory decision-making is supported through OECD (Organisation for Economic Co-operation and Development) guidance and validation frameworks, ensuring consistency, reliability, and international acceptance of NAM-derived data. QSAR, Quantitative Structure–Activity Relationship; PBPK, Physiologically Based Pharmacokinetic; AI/ML, Artificial Intelligence/Machine Learning. Figure created with Biorender.com.

This integrated approach illustrates a flexible, multi-level framework for predictive neurotoxicity and translational research (Figure 5), spanning computational prediction to *in vivo* validation. Rather than a linear hierarchy, the system operates as an iterative and modular network, where insights from *in silico* models guide *in vitro* and *in vivo* experimentation, and *in vitro* and *in vivo* data refine computational predictions. Brain organoids occupy an intermediate and integrative position, linking cellular-level mechanisms with whole-organism responses, thus enhancing both translational relevance and regulatory confidence. This dynamic integration fosters a more predictive, ethical, and mechanistically informed preclinical research paradigm.

## Conclusion

In light of the growing importance of NAMs in neuroscience as ethical, human-relevant, and efficient alternatives to traditional animal testing, we critically assessed the currently available NAMs, ranging from conventional 2D cell cultures to advanced human brain organoids, non-human organoids and zebrafish, in direct comparison to mammalian models. Moreover, their respective strengths, limitations, and roles in neurodevelopmental, pathomechanistic, and neurotoxicity research have been mapped. While each model has unique strengths, combining complementary systems is essential to overcome individual limitations, supporting more robust and scalable pipelines for early-phase screening, safety assessment, disease modeling, and drug discovery. By integrating 2D, 3D, and alternative *in vivo* models, the field can advance toward more predictive, efficient, and ethically responsible neuroscience research. Continued research, methodological refinement, and standardization will be critical to realize the full potential of NAMs.
